# Victim Sensitivity and Proposal Size Modulate the Ingroup Favoritism During Fairness Norm Enforcement

**DOI:** 10.3389/fpsyg.2021.738447

**Published:** 2021-09-29

**Authors:** Zhen Zhang, Hui Zhao, Ruixue Liu, Chunhui Qi

**Affiliations:** Faculty of Education, Henan Normal University, Xinxiang, China

**Keywords:** victim sensitivity, proposal size, ingroup favoritism, fairness norm enforcement, ultimatum game

## Abstract

People show a strong aversion to inequality and are willing to sacrifice their own interests to punish violations of fairness norms. Empirical research has found that group membership could influence the fairness judgment and norm enforcement of the individuals but has shown inconsistent findings and has not focused much on the potential moderators. Here, the two studies aimed to investigate whether victim sensitivity and proposal size moderate the impact of group membership on reactions to unfair proposals. In both studies, the participants with different victim sensitivity (low vs. high group) played the hypothetical (Study 1) and incentivized (Study 2) ultimatum game under the intragroup and intergroup condition and indicated their responses to the different proposals. Results showed that, regardless of the victim sensitivity, ingroup member is often given preferential and positive treatment. Low victim sensitive persons are more likely to accept unfair offers from the ingroup than the outgroup, while this effect was attenuated for those with high victim sensitivity, especially for highly ambiguous unfair offers (offer 6:4 in Study 1 and 8:2 in Study 2). Moreover, the ingroup favoritism score for ambiguous unfair offers was smaller for high compared with the victim sensitivity group. Taken together, the victim sensitivity, and proposal size could moderate the ingroup favoritism on responses to unfairness.

## Introduction

Fairness is a widespread social norm and plays a critical role in human interaction. People show a strong aversion to inequality (McAuliffe et al., 2017), and are willing to sacrifice own interests to sanction the violations of fairness norms (Henrich et al., 2006). The ultimatum game (UG) is a widely used task to explore human fairness. During this two-player game, a proposer offers to split money between herself/himself and a responder, who can either accept or reject it. If accepted, both the players gain, but if rejected, neither player get anything (Güth et al., 1982). The offers of 20% are typically deemed to be unfair and are rejected around half the time (Henrich et al., 2006). This enforcement of fairness norms is irrational from the economic perspective but indicates a social preference for fairness in our society (Fehr and Gächter, 2000). Alternatively, given that rejecting this unfair distribution reflects revenge and antagonism toward the provocation of the proposer, it was also described as hostile behavior or reactive aggression (Gong et al., 2017).

Intolerance of injustice and norm enforcement are critically influenced by group membership. A growing body of researchers have addressed the group bias during fairness norm enforcement and have shown mixed findings. Some evidence suggested that people are more tolerant of the unfairness of ingroup members compared with the outgroup members. For example, adults are more likely to accept unfair offers of ingroup members in soccer clubs (Reimers et al., 2017), classmates (Valenzuela and Srivastava, 2012), race stereotypes (Kubota et al., 2013), psychological hypnosis (Brüne et al., 2012), and minimal group contexts (Wang et al., 2017). Electrophysiological evidence also implied that when proposers were perceived to be unintentional, unfair offers from outgroups induced significantly larger feedback related negativity than those from ingroups in basketball teams (Wang et al., 2016) and friendship contexts (Campanha et al., 2011), which might serve as evidence of ingroup favoritism. These findings supported the social identity theory, which suggest that group attachment and positive evaluation drive individuals to favor their own group and forgive unfairness from ingroups (Tajfel, 1982; Zhang et al., 2020). Meanwhile, other studies found that people are less forgiving of ingroup perpetrators. For instance, unfair offers from ingroup members were more likely to be rejected in college affiliation (McLeish and Oxoby, 2011), race stereotypes (Mendoza et al., 2014), and minimal group contexts (Wu and Gao, 2018). In addition, the electrophysiological findings found that feedback-related negativity was more negative for unequal offers compared with equal offers in the ingroup interaction whereas it did not show differential responses to different offers in the outgroup interaction in the basketball team (Wang et al., 2016) and minimal group contexts (Wang et al., 2017), which potentially provided neural evidence for the black sheep effect. In line with norm-focused theory, these results suggested that the violation of prescriptive norms was highly unexpected and objectionable, thereby inducing more negative reactions to ingroup violators (McAuliffe and Dunham, 2016). Recent review literature has shown that most of the findings are more consistent with the social identity theory than the norms-focused theory (McAuliffe and Dunham, 2016). These mixed findings imply that group membership can affect the fairness concern and norm enforcement of individuals but leave the nature of these effects in question. Furthermore, the previous studies mostly focus on the manipulation of group membership and interactive scenarios, leaving the personality differences on fairness concern unclear. As people need to resolve the tradeoff between fairness norm and group bias in the intergroup interactions, the personality differences on intolerance of unfairness and injustice might play a critical role during this case.

To fill this gap, we turned to the concept of victim sensitivity and aimed to examine whether victim sensitivity could modulate the group bias effect on norm enforcement. Although unfairness often elicited strong reactions, people systematically differ in their victim sensitivity, which reflects their intolerance of unfairness or injustice directed toward themselves (Schmitt et al., 2005). Victim sensitivity was associated with suspiciousness, paranoia, jealousy, neuroticism (Schmitt et al., 2005), bullying, hostile attribute bias, aggression (Bondü, 2018), general anxiety, and social phobia symptoms (Bondü and Inerle, 2020). Moreover, the high victim sensitive persons possess a suspicious mindset (Gollwitzer et al., 2009), are sensitive to mean intentions (Gollwitzer et al., 2013), and easily form unjust expectancy in ambiguous situations (Maltese et al., 2016), thereby leading to uncooperative and aggressive behavior during uncertainly social situations (Fetchenhauer and Huang, 2004; Gollwitzer et al., 2005). In addition, they displayed more outgroup anger and ingroup angst (Süssenbach and Gollwitzer, 2015), rejected more unequal offers (Zhen and Yu, 2016), showed more unforgiving reactions to interpersonal transgressions (Gerlach et al., 2012), and invested less in public good and volunteer activities (Gollwitzer et al., 2009; Tham et al., 2019). In sum, victim sensitivity represents a blend of moral concerns and antisocial motives for self-protection and might not give priority to the group bias when dealing with unfair ultimatum offers. Based on these findings, we expect that the high victim sensitive persons might display weaker ingroup favoritism than those with low victim sensitivity.

Another unexplored issue was regarding whether the relationship between victim sensitivity and group bias in norm enforcement varies with the proposal size. The endowment of typical UG was 10 points, and the proposals could be fair (5:5), mild (6:4), moderate (7:3, 8:2), and extremely unfair (9:1). Without a doubt, as the proposal size increases, the proposals are perceived as more generous and fair, thereby leading to higher acceptance rates (McAuliffe et al., 2017). However, numerous studies have shown that the reactions to fair and extremely unfair offers were quite clear and consistent, while it is hard to predict the responses of people to mild and moderate offers due to their relatively high economic utility and fairness (Brüne et al., 2012; Gong et al., 2017; Wang et al., 2017). Some researchers consider that fair and extremely unfair proposals have relatively low ambiguity, while mildly and moderately unfair proposals possess higher uncertainty, and then, lead to the larger individual variation of punishment (Mendoza et al., 2014; Wang et al., 2017). During group interactions, the group-based bias of individuals would be influenced by offer size. Mendoza et al. (2014) found that participants were just more likely to reject marginally unfair offers from racial in-group than the out-group members. Moreover, the strong situation hypothesis supposed that the personality of people only manifests itself during weak situations where there is no uniform expectancy to act in a certain way (Mischel, 1977). When the proposal is mildly and moderately unfair, the individual differences of recipient's behavior would become greater. For instance, Gong et al. (2017) found that interpersonal responsibility had negative effects on the rejection behavior of responders only for moderately unfairness. Based on these observations, the attenuated ingroup favoritism for high victim sensitive persons was expected to emerge for ambiguous unfairness, for which ambiguous unfair offers from outgroup members might be perceived as acceptable and valuable.

Overall, then, this research sought to clarify the relationship between the reactions to unfairness and group bias by exploring how this relationship is modulated by the victim sensitivity of responders. Based on the previous theoretical derivation, we expected that the victim sensitive persons would be more likely to reject unfair offers, and group membership would increase the willingness of an individual to accept unfairness from ingroup than the outgroup members. In addition, we hypothesized that high, compared to low, victim sensitive persons might display weaker ingroup favoritism, especially for the ambiguous unfair offers.

## Study 1

In Study 1, we compared the behavioral reactions of high and low victim sensitive individuals to two levels of proposal size (e.g., offer 8:2 and 6:4) in the hypothetical UG when interacting with an ingroup and outgroup partner. Since offer 6:4 possessed higher uncertainty than the offer 8:2 during Study 1, we speculated that the ingroup favoritism difference between low and high victim sensitivity groups was mainly reflected in the offer 6:4. We used the hypothetical UG for two reasons: First, some research found that the results of both the hypothetical and incentivized game are usually similar (Nardi, 2018). Second, the hypothetical UG is more convenient and economic.

## Method

### Participants

A total of 440 Chinese college students (75.2% female students, M_age_ = 20.23 ± 1.34 years) who completed the Chinese version of the 10-item victim sensitivity scale developed by Schmitt et al. (2005) at first. The victim sensitivity scale is a subscale of the Justice Sensitivity Inventory (Schmitt et al., 2010), and its items were worded from the perspective of the victim (e.g., “It makes me angry when others are undeservingly better off than me”). Each item was rated on a five-point scale from 1 (absolutely disagree) to 5 (absolutely agree), and the mean score was calculated. This Chinese version showed adequate reliability and validity (Wu et al., 2014).

To explore the relationship between personality variable and behavior, a common method is comparing the behavioral differences between the high and low groups on a personality measure (Gong et al., 2017). After sorting the scores of participants on the victim sensitivity scale in descending order, 66 participants (upper 15%) were assigned to the high victim sensitive group, whereas the other 66 participants (lower 15%) were assigned to the low victim sensitive group. Then, we selected 40 subjects from each group to participate in the experiment, based on several criteria, such as they were right-handed, had normal or corrected-to-normal vision, volunteered to complete tasks, and did not report any psychiatric or neurological disorders. This sample size was determined based on a previous study (Brüne et al., 2012; Gong et al., 2017). Each volunteer was recruited for the experiment in exchange for credit and signed informed consent. The participants also completed the perspective-taking subscale of the Interpersonal Reactivity Index-China (IRI-C, Zhang et al., 2010) and the honesty–humility subscale of the HEXACO Personality Inventory-Revised (HEXACO-PI-R, Ashton and Lee, 2009), because individual differences in these variables might affect the fairness consideration and norm enforcement. The groups differed significantly on the victim sensitivity score (high vs. low: 4.11 ± 0.29 vs. 1.77 ± 0.23), *p* < 0.001, but did not differ in sex (M/F, high vs. low: 12/28 vs. 12/28), age (high vs. low: 20.23 ± 1.27 vs. 20.53 ± 1.45), perspective-taking score (high vs. low: 3.91 ± 0.66 vs. 3.64 ± 0.64), and honesty–humility score (high vs. low: 3.35 ± 0.64 vs. 3.62 ± 0.65), *p*s > 0.05. The experimental protocol was approved by the local ethics committee and adhered to the tenets of the Declaration of Helsinki.

### Procedure and Materials

The participants were told that they were going to complete a paper and pencil questionnaire about interpersonal bargaining and were tested in a separate laboratory room. The procedure consisted of three main parts, namely the group induction, the group induction check, and the UG. The paper and pencil procedure was used in the previous research on economic games (Ng et al., 2019). The experiment began with a brief instruction about the UG to distribute RMB 10 yuan. Then, the participants were asked to imagine two unknown students, one from their own university (the ingroup manipulation), and the other from another university (the outgroup manipulation). Following this, the participants answered one question about the identity of these students and completed the inclusion-of-other-in-self scale (Aron et al., 1992). Finally, the participants completed the UG with the ingroup player and with the outgroup player, in which the ingroup and outgroup member would provide two different allocations (e.g., offer 8:2 or 6:4). In other words, the present study utilized a 2 (victim sensitivity: low vs. high) × 2 (group membership: ingroup vs. outgroup) × 2 (proposal size: offer 8:2 vs. 6:4) mixed-methods experimental design, in which the victim sensitivity group was between the subjects factors, while group membership and proposal size were within the subjects factors.

### Group Membership Induction

The participants were asked to imagine meeting an unknown student from the same university and another unknown student from a different university. The former refers to the intragroup condition, and the latter refers to the intergroup condition. This group induction has been proven to be effective in previous studies (Yu et al., 2016).

### Group Membership Induction Check

To check our manipulation of the group, we asked the participants to indicate whether the interactive proposer comes from the same university (yes or no). Further, each participant completed the inclusion-of-other-in-self scale (Aron et al., 1992). The inclusion-of-other-in-self scale consisted of seven pairs of progressively interlinking circles indicating progressively closeness between the self and the other, ranging from 1 (no interlink) to 7 (extremely interlink). The participant was asked to select one out of these pairs that best described their closeness to the ingroup and outgroup player.

### Ultimatum Game

Right after the induction, each participant was instructed to imagine playing the UG against another unknown student from their own university (e.g., the intragroup interaction) and against another one from a different university (e.g., the intergroup interaction). During each game, the same hypothetical other would provide two different allocations (e.g., offer 8:2 or 6:4). The three outcome variables were measured in the UG task: (1) Allocation expectation, the amount that participants expect to receive from the proposer; (2) Acceptance possibility, the probability that participants accept each allocation with a percentage from 0 to 100% (Haselhuhn and Mellers, 2005), (3) Ingroup favoritism score, the difference in acceptance possibility for each offer between the ingroup and outgroup interaction (Schiller et al., 2014). Although both the games were hypothetical, that is, no monetary incentives or actual proposers were present, the results have been proven to be similar to those based on the direct response method (Nardi, 2018). The order of the two economic games and the different offers were counterbalanced between subjects.

## Results

### Manipulation Checks

All participants indicated correctly whether the hypothetical player came from the same or different college. Moreover, a 2 (victim sensitivity: low vs. high) × 2 (group membership: ingroup vs. outgroup) ANOVA on the inclusion-of-other-in-self scale scores yielded only a significant main effect of group membership, *F*_(1, 78)_ = 369.85, *p* < 0.001, partial η^2^ = 0.83. Participants had a closer distance to the ingroup member (*M* = 4.41, *SE* = 0.15) compared with the outgroup member (*M* = 1.49, *SE* = 0.08). These results indicated that our manipulation of the group membership was successful.

### Allocation Expectation

A 2 (victim sensitivity: low vs. high) × 2 (group membership: ingroup vs. outgroup) ANOVA on the allocation expectation displayed only a significant main effect of the group membership, *F*_(1, 78)_ = 75.48, *p* < 0.001, partial η^2^ = 0.49, with higher allocation expectation in intragroup (*M* = 4.29, *SE* = 0.22) relative to intergroup condition (*M* = 2.00, *SE* = 0.22).

### Acceptance Possibility

**Figure 1:**
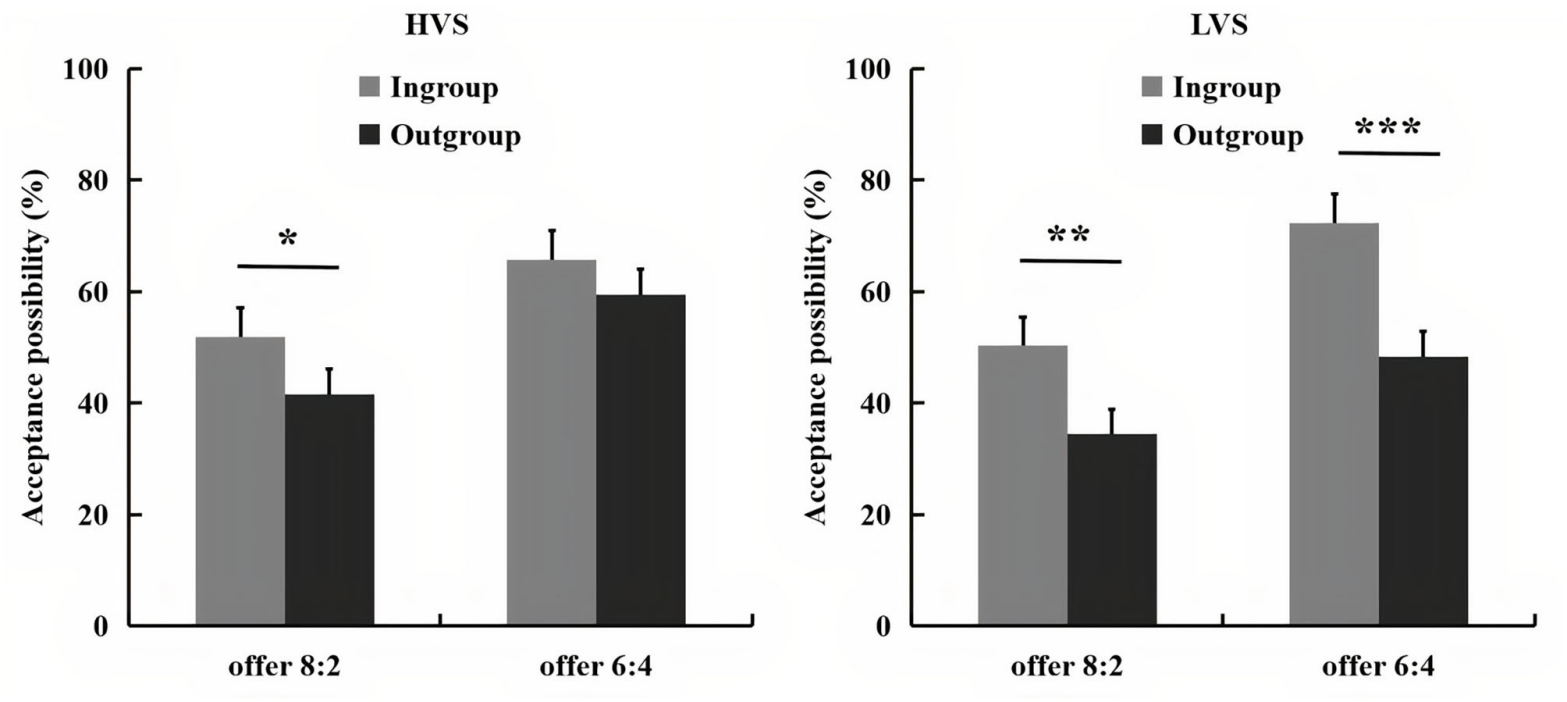
The bar graphs show the mean value of acceptance possibility for each condition. Error bars indicate SE. Asterisks indicate significant effects (*p < 0.05, **p < 0.01, ***p < 0.001).

The acceptance possibility for each offer is presented in Figure 1. A 2 (victim sensitivity: low vs. high) × 2 (group membership: ingroup vs. outgroup) × 2 (proposal size: offer 8:2 vs. 6:4) ANOVA revealed a significant main effect of group membership, *F*_(1, 78)_ = 21.10, *p* < 0.001, partial η^2^ = 0.21, with higher acceptance possibility during the intragroup (*M* = 60.00%, *SE* = 3.25) than the intergroup interaction (*M* = 45.96%, *SE* = 3.86). The main effect of proposal size was also significant, *F*_(1, 78)_ = 73.29, *p* < 0.001, partial η^2^ = 0.49, with offer 8:2 (*M* = 44.53%, *SE* = 3.57) was more likely to be rejected than the offer 6:4 (*M* = 61.43%, *SE* = 3.17).

Importantly, a significant three-way interaction among victim sensitivity, group membership, and proposal size was also found, *F*_(1, 78)_ = 6.41, *p* = 0.013, partial η^2^ = 0.08. A further simple test found that when low victim sensitivity group encountered offer 8:2, the acceptance possibility was higher for the ingroup (*M* = 50.25%, *SE* = 5.21) than the outgroup member (*M* = 34.38%, *SE* = 5.89), *p* < 0.01, whereas when low victim sensitivity group dealt with offer 6:4, the acceptance possibility was also significantly higher during ingroup (*M* = 72.23%, *SE* = 4.53) relative to the outgroup condition (*M* = 48.38%, *SE* = 5.49), *p* < 0.001. However, when high victim sensitivity group encountered with offer 8:2, the acceptance possibility was higher for ingroup (*M* = 51.88%, *SE* = 5.21) than the outgroup members (*M* = 41.63%, *SE* = 5.88), *p* = 0.032, whereas when high victim sensitivity group dealt with offer 6:4, the acceptance possibility was no longer influenced by the group membership (ingroup: *M* = 65.65%, *SE* = 4.53, outgroup: *M* = 59.45%, *SE* = 5.49), *p* > 0.05. No other main effects or interactions were significant, *p*s > 0.05.

### Ingroup Favoritism Score

**Figure 2:**
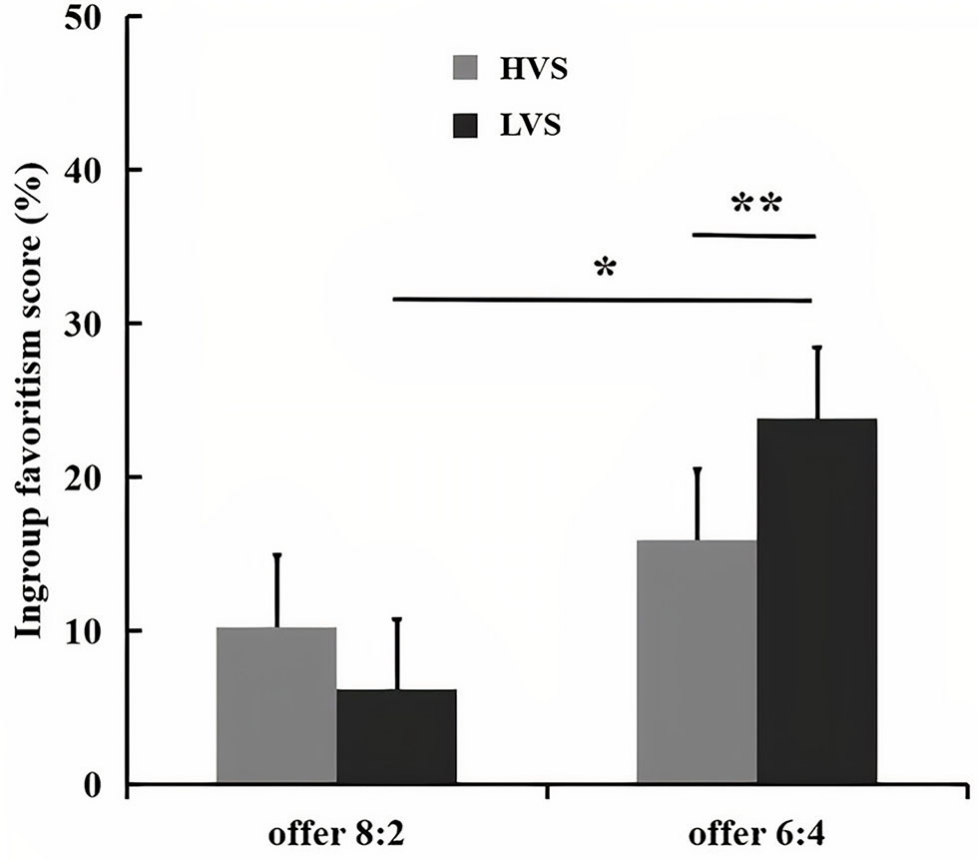
The bar graphs show the mean value of ingroup favoritism score for each condition. Error bars indicate SE. Asterisks indicate significant effects (*p < 0.05, **p < 0.01).

A 2 (victim sensitivity: low vs. high) × 2 (proposal size: offer 8:2 vs. 6:4) ANOVA only showed a significant interaction between the victim sensitivity and proposal size, *F*_(1, 78)_ = 6.41, *p* < 0.05, partial η^2^ = 0.08 (as shown in Figure 2). A further simple test displayed that low victim sensitivity group exhibited a more larger ingroup favoritism score for offer 6:4 (*M* = 23.85%, *SE* = 4.59) than for offer 8:2 (*M* = 15.88%, *SE* = 4.68), *F*_(1, 78)_ = 5.64, *p* < 0.05, whereas high victim sensitivity group exhibited no ingroup favoritism score difference between offer 6:4 (*M* = 6.20%, *SE* = 4.59) and offer 8:2 (*M* = 10.25%, *SE* = 4.68), *F*_(1, 78)_ = 1.45, *p* > 0.05. The offer-wise contrasts between the low and high victim sensitivity group revealed no significant effects for offer 8:2, *F*_(1, 78)_ = 0.72, *p* > 0.05, whereas the ingroup favoritism score of offer 6:4 was larger for low victim sensitivity group compared with the high victim sensitivity group, *F*_(1, 78)_ = 7.38, *p* < 0.01.

### Linear Regression Analysis

**Figure 3:**
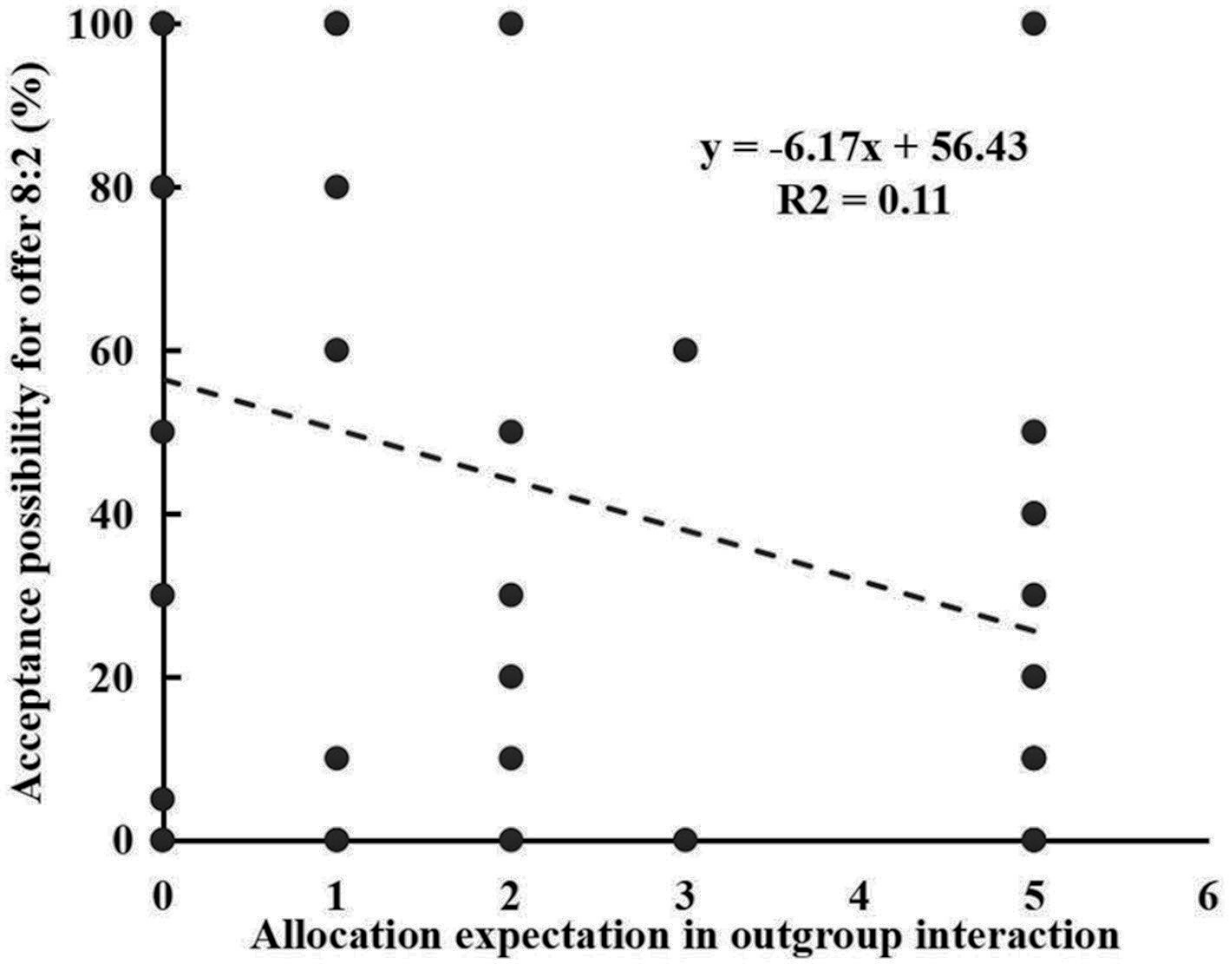
Relation between allocation expectation and acceptance possibility for offer 8:2 during the outgroup interaction.

To examine the relationship between allocation expectation and acceptance possibility of each offer during the intragroup and intergroup interaction, the linear regression analysis was conducted separately for low and high victim sensitivity groups. We only found that allocation expectation to the outgroup member was negatively correlated with the acceptance possibility of offer 8:2 for the high victim sensitivity group. In this model, the allocation expectation of high victim sensitivity group accounted for 11% of variance in the acceptance possibility of offer 8:2 during the outgroup interaction, *R*^2^ = 0.11, β = −0.33, *t* = −2.17, *p* < 0.05 (as shown in Figure 3).

## Study 2

The findings of Study 1 implied that victim sensitivity and proposal size could moderate the ingroup favoritism on responses to unfairness. To replicate the findings of Study 1 and eliminate its potential shortcomings, Study 2 aimed for a direct replication using an independent sample with the incentivized game and contains more types of the offer (5:5, 6:4, 7:3, 8:2, and 9:1). Since the offer 8:2 had been shown to produce an ~50% acceptance rate in typical UG (Camerer, 2003; Henrich et al., 2006), we hypothesized that the ingroup favoritism effect was smaller for high compared with low victim sensitivity group in the incentivized UG, especially for the offer 8:2.

## Method

### Participants

Based on the previous similar behavioral research (Gong et al., 2017), 90 Chinese college students (64.4% female students, M_age_ = 20.50 ± 1.96 years) who completed the victim sensitivity scale, the perspective-taking subscale, and the honesty–humility subscale at first, as in Study 1. After sorting the scores of participants on the victim sensitivity scale in descending order, 25 participants (upper 27%, *M* = 3.78, *SD* = 0.18) were assigned to the high victim sensitive group, whereas another 25 participants (lower 27%, *M* = 2.20, *SD* = 0.17) were assigned to the low victim sensitive group. Then, these 50 students were required to participate in the experiment to get a small amount of money. Due to some technical reasons or procedure errors, two students from the low victim sensitive group and one student from the high victim sensitive group were excluded. The groups differed significantly on victim sensitivity score (*p* < 0.001) but did not differ in sex (M/F, high vs. low: 9/15 vs. 13/10), age (high vs. low: 20.88 ± 2.40 vs. 20.43 ± 2.43), perspective taking score (high vs. low: 3.73 ± 0.80 vs. 3.51 ± 0.54), and honesty–humility score (high vs. low: 3.40 ± 0.69 vs. 3.51 ± 0.50), *p*s > 0.05.

### Procedure and Materials

The participants should complete three main parts which were similar in Study 1.

### Group Membership Induction

The participants were told that offers from players were collected from a previous behavioral experiment. During that experiment, we recruited some students from the same or different university to act as proposers of a one-shot UG and decide how to allocate 10 yuan. The participants would interact with proposers from the same and different university in the present study. The former refers to the intragroup condition, and the latter refers to the intergroup condition.

### Group Membership Induction Check

To check the manipulation of the group, we asked participants to indicate whether the interactive proposer comes from the same university (yes or no). Further, each participant completed the two items from the Overlap of Self, Ingroup, and Outgroup scale (Schubert and Otten, 2002) that were intended to measure the relation between the self and the ingroup/outgroup. Each of the items consisted of seven pairs of progressively interlinking circles indicating progressively closeness between the self and the ingroup or outgroup, ranging from 1 (no interlink) to 7 (extremely interlink). The participant was asked to select one out of these pairs that best described their closeness to the ingroup or outgroup.

### Ultimatum Game

Right after the induction, each participant was instructed to complete two separate blocks of UG, one played with unknown students from the own university (e.g., the intragroup interaction) and the other played against students from the different university (e.g., the intergroup interaction).

**Figure 4:**
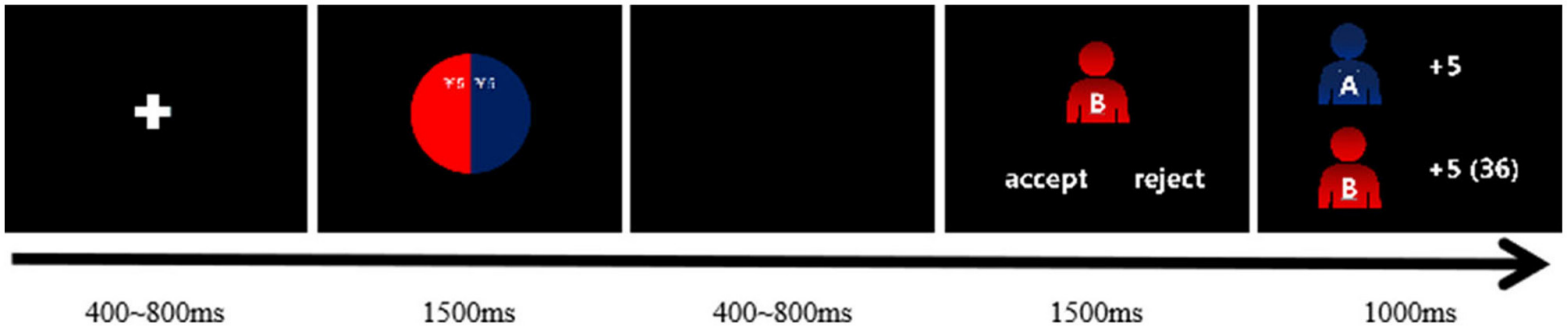
A single trial in the intergroup interaction. The responder accepts the equal proposal, and thus, both obtain the corresponding money.

Each block contains 30 trials: six each from the five proposals (1, 2, 3, 4, or 5 yuan offered). In each trial, a fixation cross was presented (400–800 ms) and followed by a divided color pie indicating the amount of the offer (1,500 ms) (as shown in Figure 4). After a black screen for 400–800 ms, participants should press a button with the index finger of his/her left or right hand to accept or reject the offer within 1,500 ms. Finally, feedback based on their responses was presented (1,000 ms), in which two colored images (red or blue) represented group membership of two players: one image (top) referring to the proposer and the other one (bottom) referring to the subject self. The subjects were randomly assigned to either a red or a yellow group and told that students from the same or different university were in the same or other group.

The block-order and the link between the buttons and decisions were counterbalanced between subjects. The trials of each block were presented in a pseudo-randomized order. Before the formal experiment, participants should complete a practice block of five trials with ingroup members. After the formal test, each participant was paid privately with 10 yuan regardless of their performance.

## Results

### Manipulation Checks

All the participants indicated correctly whether the interactive player came from the same or different college. Moreover, a 2 (victim sensitivity: low vs. high) × 2 (group membership: ingroup vs. outgroup) ANOVA on the Overlap of Self, Ingroup, and Outgroup scale scores yielded only a significant main effect of group membership, *F*_(1, 45)_ = 294.67, *p* < 0.001, partial η^2^ = 0.87. Participants had a closer distance to the ingroup (*M* = 4.49, *SE* = 0.17) compared with the outgroup (*M* = 1.62, *SE* = 0.11). These results indicated that our manipulation of group membership was successful.

### Acceptance Rates

**Figure 5:**
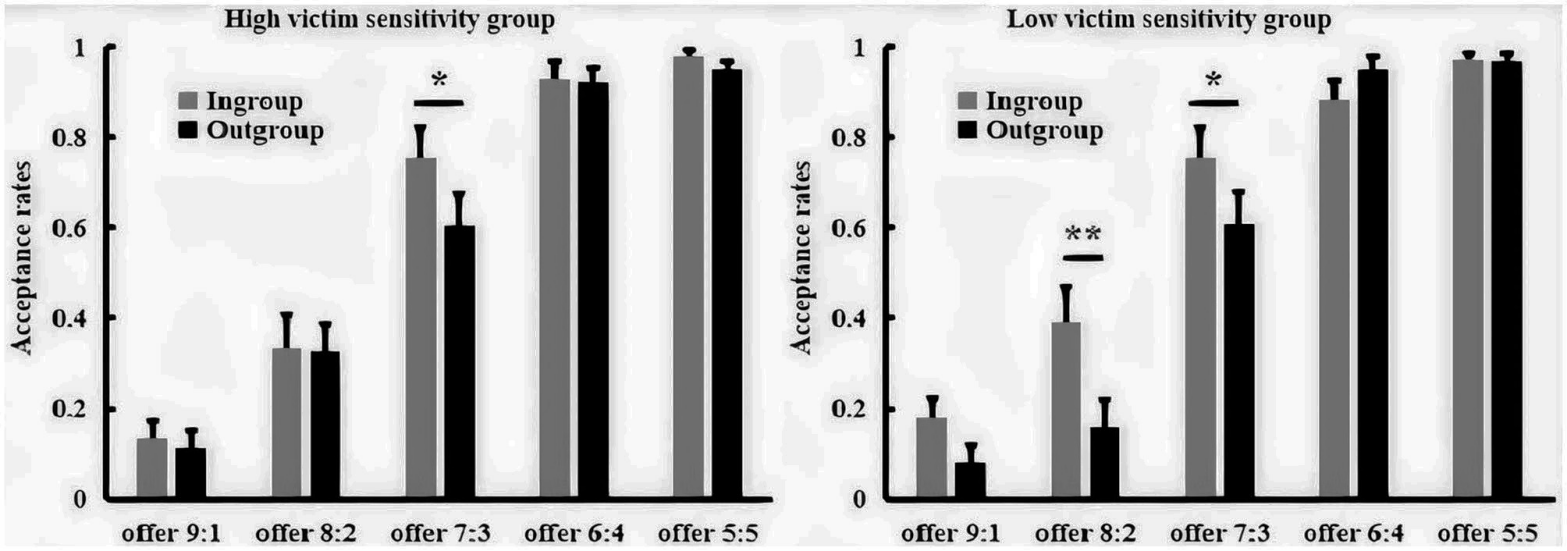
The bar graphs show the mean value of acceptance rates for each condition. Error bars indicate SE. Asterisks indicate significant effects (*p < 0.05, **p < 0.01).

The acceptance rates for each offer are presented in Figure 5. A 2 (victim sensitivity: low vs. high) × 2 (group membership: ingroup vs. outgroup) × 5 (proposal size: 9:1, 8:2, 7:3, 6:4, and 5:5) ANOVA revealed a significant main effect of the group membership, *F*_(1, 45)_ = 8.56, *p* < 0.01, partial η^2^ = 0.16, with higher acceptance rates during the intragroup (*M* = 0.61, *SE* = 0.02) than the intergroup interaction (*M* = 0.57, *SE* = 0.02). The main effect of proposal size was also significant, *F*_(4, 180)_ = 200.26, *p* < 0.001, partial η^2^ = 0.82, with the acceptance rates increase as the proposal size increases, but the acceptance rates of offer 6:4 (*M* = 0.92, *SE* = 0.02) were similar with that for offer 5:5 (*M* = 0.97, *SE* = 0.01). The interaction between group membership and proposal size was significant, *F*_(4, 180)_ = 4.46, *p* < 0.01, partial η^2^ = 0.09. The pairwise comparison displayed that the acceptance rates of offer 8:2 and 7:3 were higher for ingroup (*M* = 0.36, *SE* = 0.05; *M* = 0.75, *SE* = 0.05) than the outgroup member (*M* = 0.24, *SE* = 0.04; *M* = 0.61, *SE* = 0.05), *p*s <0.05, whereas the acceptance rates of offer 9:1, 6:4, and 5:5 were no longer influenced by the group membership, *p*s > 0.05.

Importantly, a significant three-way interaction among victim sensitivity, group membership, and proposal size was also found, *F*_(4, 180)_ = 2.88, *p* = 0.033, partial η^2^ = 0.06. A further simple test found that when low victim sensitivity group encountered offer 8:2 and 7:3, the acceptance rates were higher for ingroup (*M* = 0.39, *SE* = 0.08, *M* = 0.75, *SE* = 0.08) than the outgroup member (*M* = 0.16, *SE* = 0.06, *M* = 0.61, *SE* = 0.07), *p* < 0.05, whereas when low victim sensitivity group dealt with offer 9:1, 6:4, and 5:5, the acceptance rates were no longer influenced by the group membership, *p*s > 0.05. In contrast, when high victim sensitivity group encountered with offer 7:3, the acceptance rates were higher for ingroup (*M* = 0.75, *SE* = 0.07) than the outgroup member (*M* = 0.60, *SE* = 0.07), *p* = 0.023, whereas the group membership could not affect the acceptance rates of the other four offers for high victim sensitivity group, *p*s > 0.05. No other main effects or interactions were significant, *p*s > 0.05.

### Reaction Times

A 2 (victim sensitivity: low vs. high) × 2 (group membership: ingroup vs. outgroup) × 5 (proposal size: 9:1, 8:2, 7:3, 6:4, and 5:5) ANOVA revealed a significant main effect of group membership, *F*_(1, 45)_ = 9.66, *p* < 0.01, partial η^2^ = 0.18, with longer reaction times during intergroup (*M* = 697.98 ms, *SE* = 17.46) than the intragroup interaction (*M* = 644.50 ms, *SE* = 15.80). The main effect of proposal size was also significant, *F*_(4, 180)_ = 11.68, *p* < 0.001, partial η^2^ = 0.21, with shorter reaction times to offer 5:5 (*M* = 622.56 ms, *SE* = 15.17) than to the offer 9:1 (*M* = 675.73 ms, *SE* = 17.87), 8:2 (*M* = 693.08 ms, *SE* = 16.87), 7:3 (*M* = 699.74 ms, *SE* = 16.51), and 6:4 (*M* = 665.07 ms, *SE* = 15.10), *p*s <0.01. The interaction between the group membership and proposal size was significant, *F*_(4, 180)_ = 12.77, *p* < 0.001, partial η^2^ = 0.22. The pairwise comparison displayed that the reaction times to offer 5:5 (*M* = 595.62 ms, *SE* = 16.35) were significantly shorter for high victim sensitivity group compared with the offer 9:1 (*M* = 705.01 ms, *SE* = 21.36), 8:2 (*M* = 730.89 ms, *SE* = 23.43), 7:3 (*M* = 756.38 ms, *SE* = 22.38), and 6:4 (*M* = 701.97 ms, *SE* = 20.75), *p*s <0.01. There was no significant difference among the five offers for low victim sensitivity group, *p* > 0.05. No other main effects or interactions were significant, *p*s > 0.05.

### Ingroup Favoritism Score

**Figure 6:**
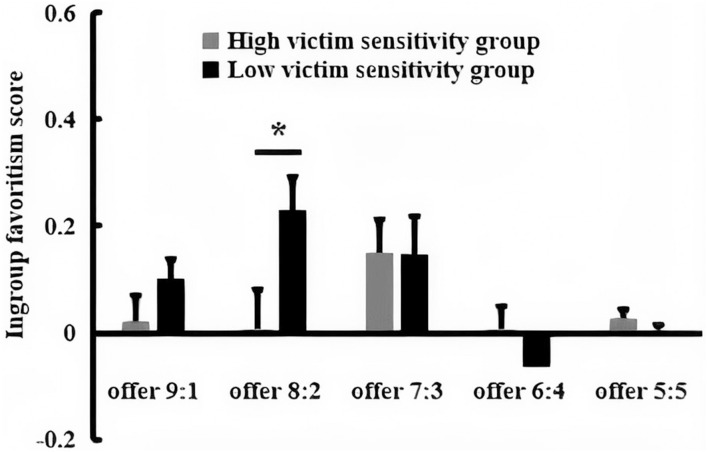
The bar graphs show the mean value of ingroup favoritism score for each condition. Error bars indicate SE. Asterisks indicate significant effects (*p < 0.05).

A 2 (victim sensitivity: low vs. high) × 5 (proposal size: 9:1, 8:2, 7:3, 6:4, and 5:5) ANOVA revealed a significant main effect of proposal size, *F*_(4, 180)_ = 4.45, *p* < 0.01, partial η^2^ = 0.09, with higher ingroup favoritism score to offer 7:3 (*M* = 0.15, *SE* = 0.05) than the offer 6:4 (*M* = −0.03, *SE* = 0.03), *p* < 0.05, and no difference among the other offers, *p*s > 0.05. The interaction between the victim sensitivity and proposal size was significant, *F*_(4, 180)_ = 2.85, *p* < 0.05, partial η^2^ = 0.06 (as shown in Figure 6). A further simple test displayed that, low victim sensitivity group exhibited a larger ingroup favoritism score for offer 8:2 (*M* = 0.23, *SE* = 0.07) than for the offer 6:4 (*M* = −0.07, *SE* = 0.04) and offer 5:5 (*M* < 0.01, *SE* = 0.02), *p*s <0.05, whereas the high victim sensitivity group exhibited no ingroup favoritism score difference among the different offers, *p*s > 0.05. The offer-wise contrasts between low and high victim sensitivity group only found that the ingroup favoritism score of offer 8:2 was larger for low victim sensitivity group (*M* = 0.23, *SE* = 0.07) compared with high victim sensitivity group (*M* = 0.01, *SE* = 0.08), *p* < 0.05, whereas there was no significant difference between the victim sensitivity group for the other four offers, *p*s > 0.05.

## Discussion

The purpose of this study was to examine whether victim sensitivity and proposal size moderate the ingroup favoritism effects on responses to unfair offers. The results of the two experiments imply three main findings. First, the manipulations of group membership in the hypothetical and incentivized game are effective, which could affect the interpersonal perception, reciprocal belief, and aggressive willingness of an individual; showing favoritism for ingroup compared with the outgroup members. Second, compare with the low victim sensitive persons, high victim sensitive persons displayed weaker ingroup favoritism, and even null effect of group membership on highly ambiguous unfair offers (offer 6:4 in Study 1 and offer 8:2 in Study 2) during group interactions; this suggests that the individuals with high victim sensitivity care more about self-interest and moral concerns, and might not give priority to the group bias, especially for highly ambiguous unfair offers. Third, the ingroup favoritism score difference between the low and high victim sensitivity group was mainly reflected in the offer 6:4 in Study 1 and the offer 8:2 in Study 2, suggesting that victim sensitivity could manifest itself during ambiguously weak situations. This pattern of results supports the hypothesis that victim sensitivity and proposal size moderate the ingroup favoritism on responses to unfair offers.

In line with the hypothesis, the perception, judgment, and behavior of a person are sensitive to the group membership during the hypothetical and incentivized game, with ingroup members often given preferential and positive treatment. Especially, comparing with the outgroup playmates, ingroup playmates are usually perceived as more intimate companions, expected to allocate higher amount, and less likely to obtain punishment for their selfishness. It is an interesting finding that people expect a better offer from the ingroup than from the outgroup members, yet they accept worst offers from the ingroup than from the outgroup members. These findings are in line with the previous studies and support the social identity theory which states that group attachment and positive evaluation drive individuals to favor their own group and forgive unfairness from the ingroups (Tajfel, 1982; McAuliffe and Dunham, 2016). For instance, the experiments suggested that the individuals clearly differentiate between partners according to their group membership, with expecting higher offers (McLeish and Oxoby, 2011) and accepting more unfair offers from ingroup members (Brüne et al., 2012; Kubota et al., 2013). Moreover, one electroencephalographic (EEG) study found that the unfair offers from ingroup compared with outgroup were evaluated as negativity and worse than expected, thereby inducing more negative feedback-related negativity (FRN), yet individuals rather tend to accept them (Wang et al., 2017). These findings imply that the early negative evaluation (as reflected by the FRN), which indexes the perceived degree of norm-violation, is overcome to behave in a norm-conform way, and such mechanism plays an important role in maintaining and stabilizing the group integrity. In addition, people more likely accepted the better offer in both the studies. Consistent with the research on UG (Güth et al., 1982; Henrich et al., 2006), the acceptance rates increase as recipient shares become more generous regardless of the group membership of the proposer, implying a social preference for being treated fairly.

Furthermore, the results found that the low victim sensitive persons are more likely to accept unfair offers from ingroup than the outgroup, while this effect was attenuated for those with high victim sensitivity, especially for highly ambiguous unfair offers. This modulatory effect seems to be driven by the null effect of group membership on the acceptance of high victim sensitivity group of highly ambiguous unfair offers (offer 6:4 in Study 1 and 8:2 in Study 2), with high victim sensitive people accepting it similarly regardless of group identity of the partner. This finding is in line with the hypothesis of the current study and could be explained by the coral features of victim sensitivity and the generosity of mild unfairness. During group-based UGs, people need to resolve the tension between the group's bias and fairness, which directly determines their reaction patterns. On the one hand, the victim sensitive individuals have a strong suspicious mindset and selfish tendencies and might not give priority to group bias when dealing with the unfair ultimatum offers, leading to the higher acceptance possibility for outgroup's unfairness and lower acceptance possibility for ingroup's unfairness. For example, the previous studies found that high victim sensitive individuals have shown more unforgiving reactions to interpersonal transgressions (Gerlach et al., 2012). Moreover, a strong suspicious mindset and sensitivity to mean intentions could easily induce hostile attribution to unfair offers, especially for the ingroups, which might lead to a certain degree of black sheep effect. Wang et al. (2016) found that when people deem that ingroup perpetrators intended to do harm to themselves, their evaluation of the behavior of ingroup perpetrators would be more negative. On the other hand, the reactions of people to ambiguous unfair offers possess larger individual variation due to its relatively high economic utility and fairness, thereby leading to ambiguous and weak situations (Gong et al., 2017). In Study 1, the acceptance of mild unfairness produces a larger value (i.e., 4 yuan out of 10) than the allocation expectation of people toward the outgroup (about 2 yuan out of 10), which makes the offer 6:4 more positive and acceptable. This comparison would lead to the higher acceptance possibility of mild unfairness, especially for high victim sensitivity individuals during intergroup interaction. For Study 2, we did not measure allocation expectation of the subject, but an ~50% acceptance rate for the offer 8:2 in the previous studies (Camerer, 2003; Henrich et al., 2006) indicated that this offer is relatively positive and acceptable, especially when interacting with the outgroups. Hence, the ingroup favoritism was attenuated for those with high victim sensitivity, and even null effect for ambiguous unfair offers during the group interactions.

Moreover, the regression analyses found that the higher high victim sensitive allocation expectation of persons, the lower the acceptance possibility of offer 8:2 during intergroup interaction. Both behavioral and neuroimaging research found that expectations of people of what they will receive was negatively associated with acceptance responses (Vavra et al., 2018), and participants might adopt the group conformity norm toward ingroup, and the expectations norm to outgroup (Wang et al., 2017). This pattern supports and extends the findings of the previous studies. A plausible interpretation of this result could be that high victim sensitive individuals might adopt the expectations norm toward the outgroup members and modulate their reaction to extremely unfairness based on the degree of expectancy violations.

In addition, people with low victim sensitivity display a larger ingroup favoritism compared with high victim sensitive person when facing mild proposal (offer 6:4) in Study 1 and moderately proposal (offer 8:2) in Study 2. As mentioned before, offer 6:4 is more ambiguous and uncertain than the offer 8:2 in Study 1. Meanwhile, offer 8:2 has been shown to produce an ~50% acceptance rate in typical UG (Camerer, 2003; Henrich et al., 2006). Therefore, the effects in both studies are similar, which reflect the personality-behavior relationship in a weak situation. This moderating effect of proposal size supports the strong situation hypothesis (Mischel, 1977), which posits that the weak situations permit individuals to behave in any way they desire, thereby facilitating the behavioral variability and the effect of personality on behavior. In other words, victim sensitivity manifests itself better among an ambiguous unfair situation, which might increase the parochialism of low victim sensitive individuals and the suspicious mindset of high victim sensitive people. This pattern is also consistent with the previous research which found a stronger personality-behavior relationship in weak situations. For example, Gong et al. (2017) found that interpersonal responsibility could negatively predict the rejection of an ambiguous offer in the hypothetical and incentivized UG.

The present study has some limitations. First, the real social categories and minimal group paradigm were two widely used methods to manipulate group identity and have some important differences in many ways (Zhang et al., 2020), thereby one could investigate whether our findings could apply to the minimal groups. Second, the present research did not examine the cognitive mechanism underlying the effect of victim sensitivity on ingroup bias during norm enforcement.

In sum, the current study shows for the first time that the differences in victim sensitivity make some individuals particularly prone to produce weaker ingroup favoritism, especially in ambiguous weak situations. These findings suggest that victim sensitivity could account for a prominent proportion of behavioral variance during group-based UGs. The results are important for the research and theories on norm enforcement, group bias, and personality-behavior relationships.

## Data Availability Statement

The data analyzed in the study can be found in the Supplementary Material.

## Ethics Statement

The studies involving human participants were reviewed and approved by Ethics Committee of Henan Normal University. Written informed consent for participation was not required for this study in accordance with the national legislation and the institutional requirements.

## Author Contributions

CQ and ZZ designed the experiment. HZ, RL, and ZZ collected and analyzed the data. CQ, HZ, and ZZ wrote the manuscript. RL and ZZ revised the manuscript.

## Funding

This study was supported by the National Natural Science Foundation of China [32000754], the Youth Foundation of the Ministry of Education of Humanities and Social Science Project of China [20YJC190030], the Philosophy and Social Science Foundation of Henan Province of China [2019CJY030], and the Humanities and Social Science Project of Henan Province of China [2020-ZDJH-152].

## Conflict of Interest

The authors declare that the research was conducted in the absence of any commercial or financial relationships that could be construed as a potential conflict of interest.

## Publisher's Note

All claims expressed in this article are solely those of the authors and do not necessarily represent those of their affiliated organizations, or those of the publisher, the editors and the reviewers. Any product that may be evaluated in this article, or claim that may be made by its manufacturer, is not guaranteed or endorsed by the publisher.
